# Receptors of Hypothalamic-Pituitary-Ovarian-Axis Hormone in Uterine Myomas

**DOI:** 10.1155/2014/521313

**Published:** 2014-06-22

**Authors:** Danuta Plewka, Jacek Marczyński, Michał Morek, Edyta Bogunia, Andrzej Plewka

**Affiliations:** ^1^Department of Cytophysiology, Chair of Histology and Embryology, Medical University of Silesia, Medykow 18 Street, 40-752 Katowice, Poland; ^2^Department of Proteomics, Medical University of Silesia, Jednosci Street 8, 41-200 Sosnowiec, Poland

## Abstract

In this study the expression of GnRH, FSH, LH, ER-*α*, ER-*β*, and PR receptors was examined in uterine myomas of women in reproductive and perimenopausal age. In cases of GnRH and tropic hormones a membranous and cytoplasmic immunohistochemical reaction was detected, in cases of ER-*α* and PR the reaction was located in cell nucleus, and in the case of ER-*β* it manifested also a cytoplasmic location. In some of the examined cases the expression was detected in endometrium, myocytes, and endothelium of blood vessels, in uterine glands and myoma cells. In myometrium the level of GnRH and LH receptors increases with age, whereas the level of progesterone and both estrogen receptors decreases. In myomas of women in reproductive age, independently of their size, expression of GnRH, FSH, and LH receptors was more pronounced than in myometrium. In women of perimenopausal age, independently of myoma size, expression of LH and estrogen *α* receptors was higher while expression of GnRH receptors was lower than in myometrium. FSH receptor expression was not observed. Expression of estrogen receptor *β* was not affected by age of the woman or size of myoma. Analysis of obtained results indicates on existing in small myomas local feedback axis between GnRH-LH-progesterone.

## 1. Introduction

Myomas represent the most frequently manifested nonmalignant tumours of female generative system, developing from smooth muscles. Depending on the sources of information, their prevalence ranges from 20% to 65%, to increase with age and reach the peak values during the fourth and fifth decade of life [1–6]. Myomas are seldom manifested before pubescence and tend to regress in the postmenopausal period [7, 8].

For many years now vast amounts of data have been collected on the risk factors contributing to development of myomas. Nevertheless, no unequivocal conclusion can be drawn for a single factor, as most frequently they occur together [9]. Therefore, taking into account that many of them involve the so-called modifiable factors, it becomes apparent that their interpretation sets forth numerous difficulties.

The epidemiological studies have revealed positive correlation between the age of the patients and manifestation of myomas [10]. Development of myomas increases drastically throughout the subsequent decades of age, reaching 60% within the range between 40 and 60 years [11, 12]. Relation between obesity and augmented risk of myoma has now been well documented. It reflects alterations in hormonal metabolism, pointing to the primary role of increased conversion of suprarenal androgens to estrone in the adipose tissue.

Gonadoliberin (GnRH) represents a hormone releasing gonadotropins, defined also as a factor to release the luteinising hormone (LH-RH or luliberin) and/or a hormone which releases the folliculotropic hormone (FSH-RH). It represents decapeptide, secreted by axons of neurons in the hypothalamic arcuate nucleus, with terminals close to the hypophyseal portal system.

FSH is a glycoprotein responsible for reproduction in both genders. It is indispensable for development of gonads, pubescence, and production of gametes during the reproductive period. In women, FSH at the follicular phase of the menstrual period stimulates the growth and recruitment of primordial follicles in ovaries and prevents against cell apoptosis in the antral follicles. Due to the effect of FSH, the dominant ovarian follicle secretes estradiol, which upon the negative feedback effect on hypophysis and hypothalamus induces reduction in FSH level [13, 14]. The receptor for FSH (FSHR) is a protein membrane receptor of the G protein-coupled receptor family. In the mechanism of FSH receptor function, the pathway activating protein kinase C and the activating calcium channels also are worth attention, even if they probably involve the secondary activation mechanisms [15]. In the human body the receptor is present in the genital organs exclusively.

Both in women and in men, LH is responsible for reproduction. In females, when the ovarian follicle matures due to the effect of FSH, estradiol, upon positive feedback, induces sudden secretion of LH, lasting from 1 to 2 days, responsible for induction of ovulation. Moreover, LH initiates transformation of the ovarian follicle remnants into corpus luteum which, through the release of progesterone, prepares uterus for implantation of the fertilized ovum cell. In addition, LH stimulates theca cells to produce androgens—the precursors of estrogens. For several years, LH receptor was thought to be located exclusively in some selected gonadal cells. Undoubtedly, such cells include Leydig cells of male gonad as well as in ovaries the theca cells, interstitial cells, granulosa cells, and cells of the corpus luteum [16]. Studies on location of mRNA for LH receptor pointed to its expression in uterus and oviducts in a number of laboratory animals and in humans [17].

Estrogen receptors (ER) involve hormones-activated transcription factors, belonging to the superfamily of nuclear receptors. Recognized at present, two types of estrogen receptors, *α* and *β*, do not represent isoforms but distinct proteins, coded by two separate genes, ESR1 and ESR2. However, the two receptors show high similarity in the amino acid composition and in the domain structure, each responsible for binding of the ligand, dimerization, binding of DNA, and activation of transcription [18].

Progesterone receptor (PR) represents an intracellular, ligand-activated transcription factor, member of the superfamily of nuclear receptors. In line with the tradition, estrogen is thought to represent the primary promoter of myoma growth. Recent biochemical, histological, and clinical evidence suggests a significant role of progesterone in the growth of uterine myomas; in line with these studies, progesterone and progesterone receptors may increase proliferative activity in myomas.

This study aimed at analysis of the hormonal environment in uterine myomas and in the healthy tissue of the female organ in a search for a potential conservative treatment. Therefore, an attempt was made to establish possible differences in the amount and distribution of sex hormone receptors in myomas, isolated from women of various age, as compared to the controls. We also attempted to demonstrate any differences in the amount and location of ER-*α* and ER-*β* and in the receptors for FSH, LH, and GnRH in the sampled material. It was also significant to recognize distribution of the studied receptors within myoma, since it has been suggested that the size of a myoma affects distribution of protein expression and, therefore, we examined expression of selected receptors in small and large myomas and defined the effect of female age on the expression level of the receptors.

## 2. Materials and Methods

### 2.1. Patients

The study comprised 40 patients with myomas at the reproductive age (below 45th year of age, FSH < 30 mIU/mL; samples collected during the follicular phase of the menstrual cycle) and 40 patients with myomas at the perimenopausal age (45–55 years, FSH > 30 mIU/mL). Inclusion criteria involved myoma detected by USG, qualification to hysterectomy, and informed consent to the planned study. The exclusion criteria included therapy with any medication, including hormonal drugs for at least 3 months before inclusion to the studies, neoplastic disease, endometrial hypertrophy, metabolic and systemic disturbances, and nicotinism. Evaluation included only the material from uteruses with one large myoma or one large and a few small myomas.

Myometrial samples (control groups) were taken from 10 women (<40 years old) undergoing hysterectomies for ovary tumors and 10 older women (>52 years old) undergoing hysterectomies for uterine prolapse.

The investigative procedures were approved by the local Medical Bioethical Commission.

Group 1 is denoted by “control group of reproductive age women”: myometrium of young women, in whom hysterectomy was performed for reasons other than uterine leiomyomas (*n* = 10). Group 2 is denoted by “small myomas of reproductive age women”: leiomyomas of <3 cm in diameter (*n* = 20). Group 3 is denoted by “large myomas of reproductive age women”: leiomyomas of >5 cm in diameter (*n* = 20). Group 4 is denoted by “control group of perimenopausal age women”: myometrium of perimenopausal age women, in whom hysterectomy was performed for reasons other than uterine leiomyomas (*n* = 10). Group 5 is denoted by “small myomas of perimenopausal age women”: leiomyomas of <3 cm in diameter (*n* = 20). Group 6 is denoted by “large myomas of perimenopausal age women”: leiomyomas of >5 cm in diameter (*n* = 20).

### 2.2. Histology

Tissue samples were fixed in 10% (v/v) solution of buffered formalin for 24 h at 4°C, then dehydrated, cleared in xylenes, and embedded in paraffin.

### 2.3. Immunohistochemical Studies

Paraffinsections (5 *μ*m) were mounted on silane-coated slides, dewaxed, and rehydrated. The sections were treated with 10 mM citrate buffer, pH 6 (30 min at 95°C), or Tris-EDTA pH 9 (45 min at 95°C) in water bath for antigen retrieval, then treated with 1,5% (v/v) H_2_O_2_ in methanol for 20 min for quenching of endogenous peroxidase activity, and equilibrated in 10 mM PBS-0,1% v/v Tween 20, pH 7,5. Nonspecific binding was reduced by incubation in 1% BSA for 60 min. Next, the slides were incubated with rabbit anti-ER-*α* (Abcam Cambridge, USA), rabbit anti-LHR (Santa Cruz Biotech, Inc., USA), rabbit anti-GnRHR (Abcam, Cambridge, USA) and goat anti-FSHR (Santa Cruz Biotech, Inc., USA) polyclonal antibodies or mouse anti-ER-*β* and mouse anti-PR monoclonal antibodies in a humidified chamber for 22 h at 4°C. After washing in PBS-Tween 20 the sections were incubated with biotinylated goat anti-rabbit, horse anti-mouse, and rabbit anti-goat immunoglobulins (Vector Laboratories Inc., Burlingame, USA), respectively, for 30 min, and next with avidin-biotinylated peroxidase complex (Vector) for 30 min. The bound antibodies were visualised with diaminobenzidine (DAB) and H_2_O_2_ in PBS, pH 7,5 according to the suppliers' instructions (Vector). Finally, the tissues were stained with Gill's hematoxylin, dehydrated, and cover-slipped. Negative controls were performed by substituting the primary antibodies with rabbit IgG, mouse IgG, and goat IgG, respectively.

### 2.4. Archives

Photographic documentation was prepared using a light microscope with a photographic attachment. Every reaction was documented by 10 photographs under 200x magnification (×20 lense and ×10 ocular) using an Eclipse E200 microscope with DS-Fi1 digital camera (Nikon).

### 2.5. Optical Density Analysis

In each positively stained cell, the intensity of staining was measured as the optical density of the reaction product, with the image analysis program NISAR the average optical density was calculated for each analyzed area. Three sections were analyzed for each of the proteins evaluated and for every patient. Ten fields were examined within each section. Finally, the arithmetic mean and standard deviation were calculated.

### 2.6. Statistical Analysis

Normal distribution of data was confirmed by the Kolmogorov-Smirnov test. Results are presented as a mean ± standard deviation. The Student's *t*-test was performed. A *P* value <0.05 was considered to be statistically significant.

## 3. Results

### 3.1. GnRH Receptor

**Figure 1:**
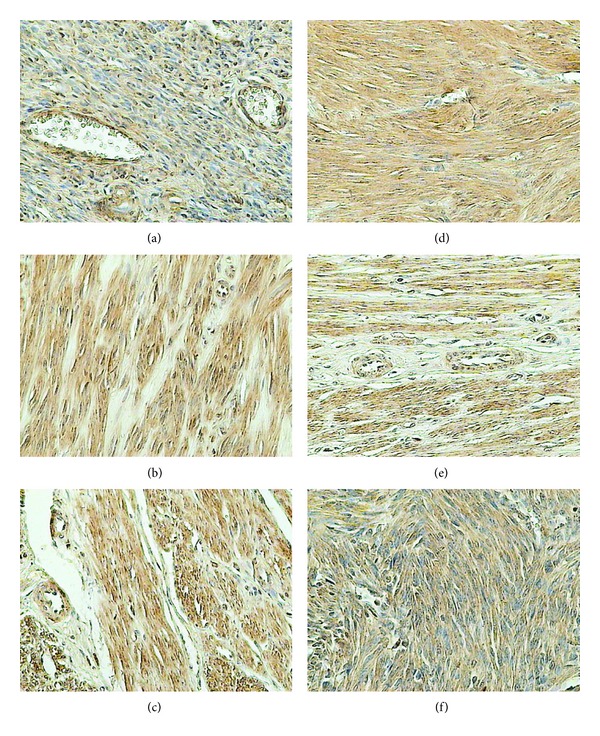
Immunohistochemical staining of uterine samples from reproductive (a)–(c) and perimenopausal age women (d)–(f) with rabbit anti-GnRH polyclonal antibodies. (a), (d) myometrium of control groups; (b), (e) small myomas; (c), (f) large myomas.

GnRH receptor demonstrated the membranous and cytoplasmic location. In the control group it was located in the endometrial epithelium, myocytes of blood vessels, myometrium, and cells of the endometrial connective tissue stroma (Figure 1). On the other hand, in all the examined groups, the reaction was located in myoma cells and in myocytes of the blood vessels. In case of myomas samples of endometrium were not available.

In reproductive women, expression of GnRH receptor was more pronounced than that in the control irrespective of the myoma size (Table 1). In perimenopausal women, in both of the examined groups, the expression of the receptor was lower than that in the controls. Expression of the receptor in perimenopausal women of the control group was higher than that in the control group of women at the reproductive age. Analogous analysis of the examined groups pointed to the comparable expression of the receptor demonstrated in small myomas. In the group with large myomas the expression level of GnRH receptor in perimenopausal women amounted to 75% of the level documented in younger women.

### 3.2. FSH Receptor

**Figure 2:**
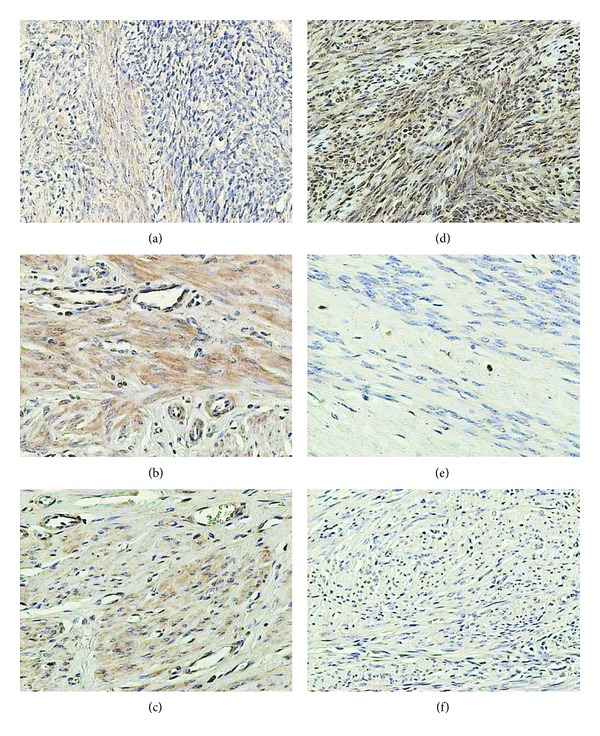
Immunohistochemical staining of uterine samples from reproductive (a)–(c) and perimenopausal age women (d)–(f) with goat anti-FSH-R polyclonal antibodies. (a), (d) myometrium of control groups; (b), (e) small myomas; (c), (f) large myomas.

Within the uterus, FSH receptor was manifested in the endothelium of blood vessels, the smooth muscle cells of blood vessels and myometrium, and the myoma cells (Figure 2).

In both control samples, the immunohistochemical reaction was manifested in only 10% of the myometrial cells and, as mentioned above, in myoma cells but only in women at the reproductive age. In small myomas it was observed in 50% and in large myomas in 25% of myoma cells (Table 1).

Independently of myoma size, expression of FSH receptor in reproductive women was higher than that in the controls. In large myomas the expression was lower than that in small myomas.

In both control groups, the expression of the receptor persisted at a similar, relatively low level. In myomas of women at the perimenopausal age the level of FSH receptor expression proved to be undetectable.

### 3.3. LH Receptor

**Figure 3:**
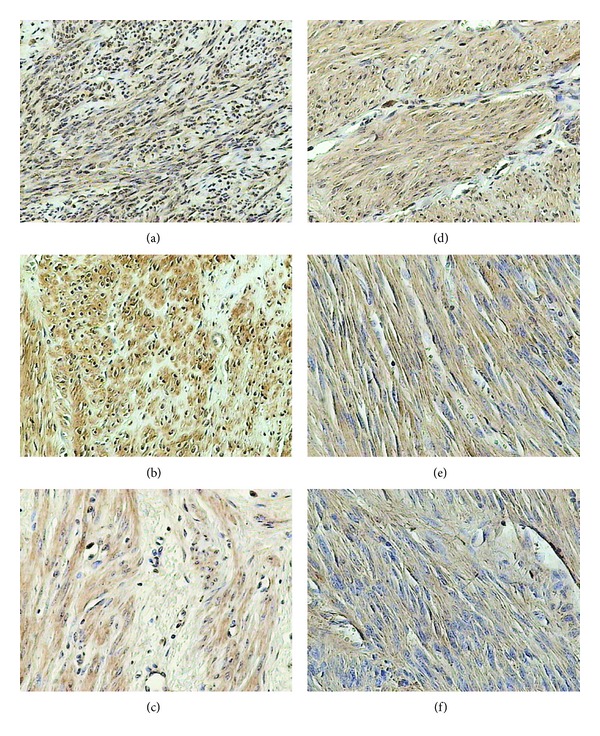
Immunohistochemical staining of uterine samples from reproductive (a)–(c) and perimenopausal age women (d)–(f) with rabbit anti-LH-R polyclonal antibodies. (a), (d) myometrium of control groups; (b), (e) small myomas; (c), (f) large myomas.

The immunohistochemical reaction for LH receptor was detected in cytoplasm and in the cell membrane. It was located in endothelial cells and in myocytes of the blood vessels. In the control groups the reaction was detected also in the myometrial cells; in the examined groups it was noted also in the myoma cells (Figure 3).

In reproductive women the expression was detected in 40% cells in the controls and in 90% cells of myomas. In the older women, in respect to both the intensity of the reaction and the number of positive cells, the population of stained cells amounted to 40% in the controls and 60% in the myomas (Table 1).

In women at the reproductive age the expression of LH receptor was higher than in the controls, irrespectively of the myoma size. In the perimenopausal group, the levels of LH receptor expression were the same, irrespectively of the myoma size.

Expression of LH receptor in the control group of perimenopausal women was higher than in control group of the reproductive women. Analysis of alterations in the level of the receptor expression in the examined groups pointed to the reduced expression at the perimenopausal age.

### 3.4. ER-*α* Receptor

**Figure 4:**
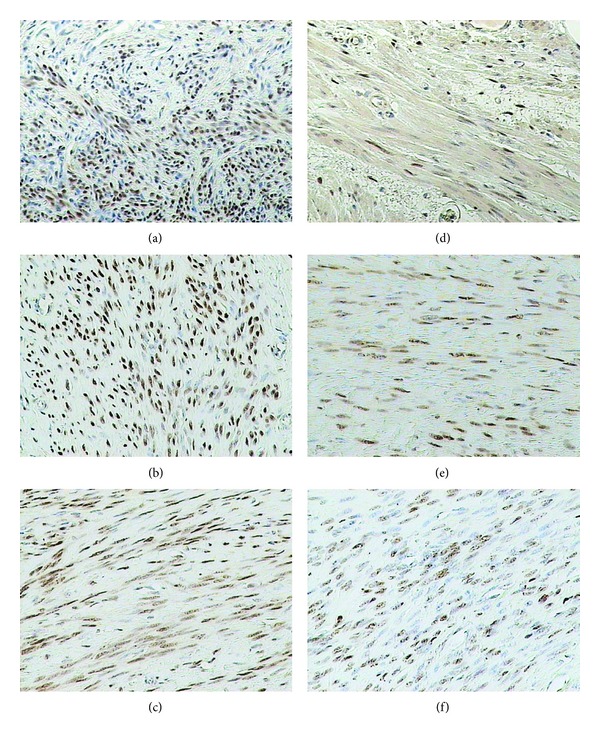
Immunohistochemical staining of uterine samples from reproductive (a)–(c) and perimenopausal age women (d)–(f) with rabbit anti-ER-*α* polyclonal antibodies. (a), (d) myometrium of control groups; (b), (e) small myomas; (c), (f) large myomas.

In the control group of women at the reproductive age, a nuclear immunohistochemical reaction was detected in cells of the uterine glands, in 70% of cell nuclei in the endometrial connective tissue and in 50% of the myocyte cell nuclei (Figure 4).

In small and large myomas of the reproductive women, expression of the receptor was more pronounced, including, respectively, 90% and 80% of myoma cells. In perimenopausal women, the nuclear reaction was manifested in 60% cells (Table 1).

Analysis of ER-*α* receptor expression in women at the reproductive age disclosed no differences among all the examined groups. In women at the perimenopausal age expression of ER-*α* receptor in both examined groups was higher than that in the controls. Irrespectively of the female age, no differences were disclosed between the groups with myomas manifesting different size.

Expression of the receptor in the control group of perimenopausal women was significantly lower than that in the control group of the reproductive women, amounting to only 60% of the level noted in the younger women. In both examined groups of women at the perimenopausal age, lower levels of ER-*α* receptor expression were detected.

### 3.5. ER-*β* Receptor

**Figure 5:**
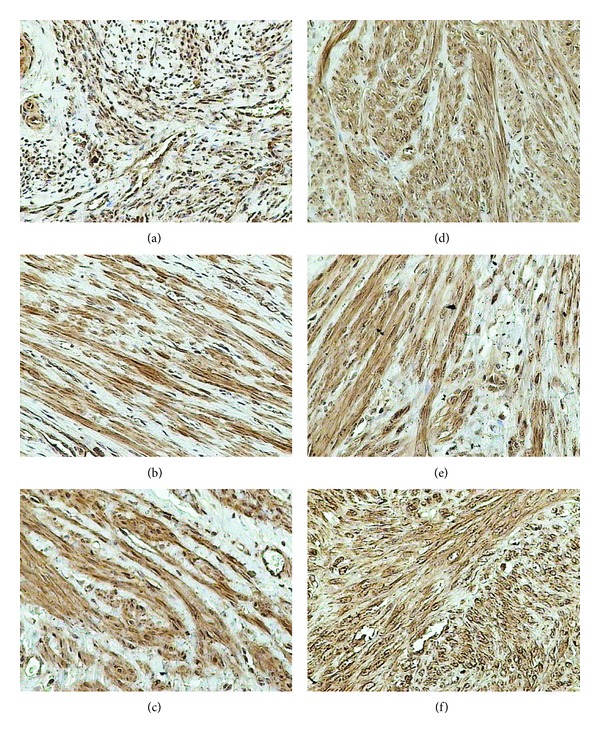
Immunohistochemical staining of uterine samples from reproductive (a)–(c) and perimenopausal age women (d)–(f) with mouse anti-ER-*β* monoclonal antibodies. (a), (d) myometrium of control groups; (b), (e) small myomas; (c), (f) large myomas.

In the control group of reproductive women, the immunohistochemical reaction was detected only in the cell nuclei. It was manifested in vascular endothelium and vascular myocytes, in 10% of the uterine gland cells and 10% of myocytes (Figure 5).

In small and large myomas of women at the reproductive age the nuclear reaction was detected in 90% of cells while the cytoplasmic reaction was noted in 50% of cells.

In the control group of perimenopausal women the cytoplasmic reaction was detected in all cells, while 70% manifested also the nuclear reaction. In perimenopausal women with small myomas the nuclear reaction was detected in 90% of cells and the cytoplasmic reaction in 50% of cells. In the group with large myomas the nuclear reaction was detected in 50% of cells and the cytoplasmic reaction in 70% of cells.

In the analysis of ER-*β* receptor expression in women at the reproductive age and in the perimenopausal women, no differences were disclosed between the examined groups. Also, irrespectively of the female age, no differences were detected between the groups with myomas of distinct size.

Expression of the receptor in the control group of women at the reproductive age was significantly higher than that in the perimenopausal controls. In the group of older women the level of ER-*β* receptor expression amounted to around 75% of the level demonstrated in the younger women. In both groups of perimenopausal women, lower levels of ER-*β* receptor expression were detected (Table 1).

### 3.6. Progesterone Receptor

**Figure 6:**
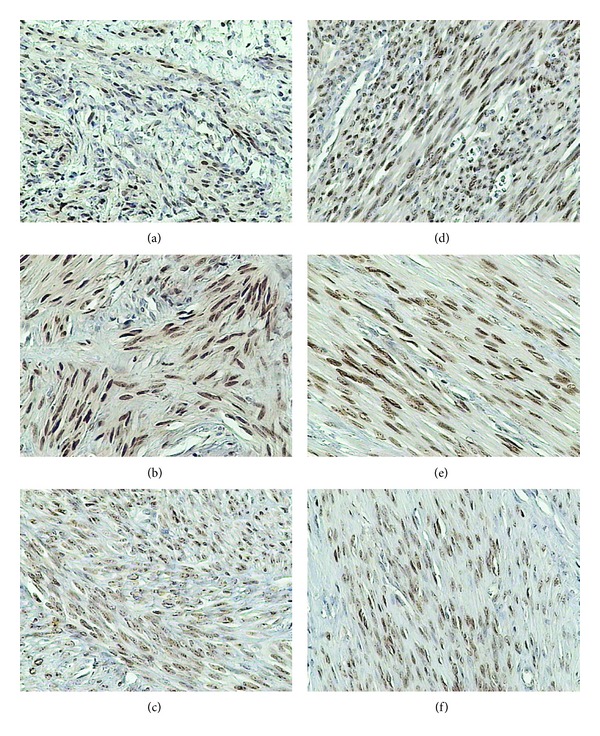
Immunohistochemical staining of uterine samples from reproductive (a)–(c) and perimenopausal age women (d)–(f) with mouse anti-PR monoclonal antibodies. (a), (d) myometrium of control groups; (b), (e) small myomas; (c), (f) large myomas.

In the control group of reproductive women, the nuclear immunohistochemical reaction was detected in cells of the uterine glands, the endometrial connective tissue, and only 10% nuclei of the smooth muscle cells (Figure 6).

In myomas of small and large size, in women at the reproductive age, high expression of progesterone receptor was detected involving, respectively, 95% and 75% of myoma cells. In perimenopausal women, the nuclear reaction in the control group included 90% of cells. In the examined groups with small or large myomas the reaction was detected in 85% and 60% of cell nuclei, respectively.

In reproductive women, expression of progesterone receptor was higher in small myomas. In large myomas expression of the receptor was reduced to around 75% of the control group level. In the group of perimenopausal women analogous changes were detected, with higher increase in expression in the group with small myomas and more pronounced decrease in the group of large myomas. Analysis of differences in expression of the receptor demonstrated lower levels in women with large myomas than in the group with small myomas (Table 1).

Expression of the receptor in the control group of reproductive women was significantly higher than in the control group of perimenopausal women group. Analogous analysis conducted for the evaluated groups showed that expression of the receptor detected in small myomas of women in perimenopausal age amounted to around 80% of the expression found in the younger group of patients. In large myomas the expression level of progesterone receptor detected in perimenopausal women amounted to only 60% of the value noted in the young women.

## 4. Discussion

Numerous publications available prove that every type of myoma treatment continues to induce interest of investigators, focused on results of treatment, complications, and favourable distant sequels. Detailed recognition of relationships which control development and growth of myomas may promote design of a new or improved manner of therapy in the morbid unit.

The literature available presents investigations which evaluated expression of receptors for hormones both in normal uterine tissues and in the uterine myomas. Our study focused on differences in expression of receptors in myomas, dependent on their size and age of the patients. First of all, we have evaluated expression of GnRH receptor, representing a superior hormone in hormonal control of female reproductive organs.

Already at the turn of the centuries, the presence of GnRH-binding sites was demonstrated in uterine myomas, suggesting direct effect of GnRH analogues upon the uterine tissues [19]. In studies of Hall et al. [20], related to women at the menopausal age, in the absence of a feedback resulting from deficit and disturbed rhythm of ovarian steroid hormones secretion, not only the elevated level of plasma gonadoliberin was detected but also changes in its secretion rhythm, appearing as decreased frequency and amplitude [20]. It may be assumed that higher expression of GnRH receptor, detected in our investigations in the control group of women at the perimenopausal age, as compared to the control group of the reproductive women reflected just the variability in plasma gonadoliberin levels. On the other hand, in tissues of the uterine myomas in perimenopausal women, decreased expression of GnRH receptor was detected, although expression was reduced significantly only in large myomas.

In hormone-dependent neoplastic cell lines, application of GnRH agonists resulted in desensitization due to the decreased amount of the membrane receptor and its internalization [21]. Similar situation may be thought to develop upon the increase in GnRH secretion during the perimenopausal period and this could probably account for the decreased expression of the receptor in neoplastic myoma cells. A number of respective in vitro experiments were performed. In cellular cultures of uterine myomas treated with buserelin (an analogue of GnRH), Kobayashi et al. [22] observed inhibition of cell proliferation, the extent of which was dependent on the dose of the drug. Moreover, they noted inhibition of cell aggregation, present in cell cultures with no GnRH analogues. In another investigation, Wang et al. [23] evaluated proliferation to estimate proliferating cell nuclear antigen (PCNA), a marker of apoptosis and mRNA for GnRH receptor. Following treatment with GnRH analogue, they detected an analogue dose-dependent inhibition of proliferation and intensification of apoptosis.

Subsequently, we have evaluated expression of FSH receptor, the ligand of which, the hormone of the consecutive level in the hypothalamus-hypophysis-gonadal axis, plays a significant role in functions of the female genital organ. Among others, it was demonstrated that FSH by itself exerted no mitogenic effects on the uterine muscle cells but together with hCG or LH stimulation, it induced moderate hyperplasia of around 30% of the cultured cells [24]. Moreover, the presence of mRNA for FSH receptor was demonstrated. In turn, in studies on hypophysectomized mice stimulated with estrogens, with or without exogenous FSH, Wang and Greenwald [25] demonstrated increased weight of ovaries and uterus, more pronounced in the group treated with the substances in parallel. The authors suggested that FSH plays an additional, if not a primary, role in stimulation of myoma cell proliferation and that its receptor may undergo stimulation even in the absence of FSH itself. Unfortunately, it still remains unknown whether it involves stimulation by locally secreted FSH or cross-stimulation by other substances in circulation.

References are available pointing to the presence of FSH receptor in the uterine muscle cells, depending on the stage of the menstrual cycle in the reproductive women; the presence of the receptor was confirmed in the proliferative and secretory phases. Significantly increased expression of mRNA for the receptor was documented in the endometrium at the secretory phase [26]. In the literature available, expression of FSH receptor in the uterine myomas was sporadically described, mainly in the aspect of application of gonadoliberin analogues. During the perimenopausal period the hormonal insufficiency of ovaries with the subsequent estrogen deficiency leads to the absence of inhibition, targeted at hypothalamus and hypophysis, resulting in high plasma concentrations of FSH and LH as well as their periodic oscillations.

Our studies, evaluating FSH receptor, demonstrated that its expression in reproductive women is significantly higher in myomas than in normal uterine muscle cells. Analogous observation was made by other groups [27], demonstrating also the absence of FSH receptor expression in ovaries of perimenopausal women. This may suggest that it reflects disturbed secretion of FSH, which, in studies on both animal models and humans, manifests a more irregular secretion mode than that of LH [28].

At the subsequent stage of our studies, expression of LH receptor was evaluated and proved to be higher than the control values in both age groups, independently of the myoma size. In groups examined at the reproductive age, a significantly lower expression has been detected in large myomas, as compared to the small ones. In perimenopausal women no significant differences were detected between the examined groups. Considering the control and manifestation of LH receptor, its direct link with action of FSH should be kept in mind. In ovaries of pigs in large, maturing follicles, the size of which depended directly on FSH at subsequent stages of maturation higher amounts of LH receptors, were detected more than in smaller follicles. In studies conducted on rats, administration of exogenous FSH was followed by the radical increase in LH-binding sites in cells of the ovarian granulosa layer. The situation becomes additionally complicated by the already widely recognised desensitization of LH receptors, manifestation of which needs just the preovulatory peak of LH secretion [29, 30].

Literature of the subject reports on a few investigations related to estimation of LH receptor in the uterine myomas. Singh et al. [31] conducted such estimations revealing expression of LH receptors lower than in normal myometrium. In the study most of the material samples were sampled from women at the reproductive age. On the other hand, in case of women close to the perimenopausal age only one of the samples showed results consistent with that obtained by us, manifesting the increased expression of LH receptor.

While evaluating the reduced expression of FSH and LH receptors in large, as compared to small, myomas in reproductive women, attention should be paid to the significance of extracellular matrix in development of myomas [32]. Assuming that the increase in myoma diameter is accompanied by the increase in the matrix, this may result in an absolutely reduced amount of the receptor per microscope visual field. An additional argument supporting such interpretation involves analysis of the immunohistochemical reaction, which in case of FSH and LH receptors has been located in the vascular endothelium, the uterine myocytes, and the myoma cells. It is likely that for this reason, the expression of LH receptor was significantly higher than that in the controls, while comparison between the experimental groups showed values slightly lower in large myomas.

In analysis of LH receptor expression in perimenopausal women, we had originally expected to obtain results analogous to those in case of FSH receptors, that is, lower or absent expression. Nevertheless, expression of LH receptors was significantly higher than that in the controls. Perhaps this reflected the already mentioned secretion of LH, which manifested lower variability [33] and, consequently, receptor desensitization of the cells was reduced.

The recent study aimed also at evaluation of expression of estrogen receptors *α* and *β*. In our results, expression of the estrogen receptor in women at the reproductive age persisted at the similar level in the controls and in the examined groups. In perimenopausal women, expression was similar except for the control group where it was significantly lower. Upon estimations of expression manifested by estrogen receptor *β* in reproductive and perimenopausal women, no significant differences were detected between the two groups of patients. Similar to receptor *α*, significantly reduced expression of receptor *β* was disclosed in the control group of the perimenopausal women, as compared to the control group of reproductive women.

Estrogens and their receptors play an important role in etiopathogenesis of the uterine myomas [34, 35]. Both *α* and *β* receptors are present in normal myometrium and in the uterine myoma tissues. In the studies performed so far, relatively extensive differences were detected, even within different tumours originating from the uterus of the same patient [36, 37]. This illustrates that myomas are characterized by extensive heterogeneity, which may be the cause of occasionally observed, even marginally different results published.

In their studies, Englund et al. [34] demonstrated overexpression of estrogen receptors in myomas, as compared to normal myometrium. They also found that the expression both in myomas and in myometrium was lower than during the proliferative phase upon increased concentration of progesterone at the secretory phase. It was suggested that progesterone reduced the amount of estrogen receptors in myomas and the myometrium.

Results opposite to ours were obtained by Regidor et al. [38], showing increased contents of estrogen receptors in myomas of patients treated with GnRH analogues. The results proved consistent with certain clinical observations. In a situation when myomas are not resected after the pharmacological treatment and GnRH analogues are discontinued, rapid myoma growth follows and the clinical signs/symptoms relapse. Similar results were obtained by Fernandez-Montoli et al. [39] where untreated women additionally showed the elevated concentration of estrogen receptors in myomas, as compared to myometrium. Such increased expression of estrogen receptors in myomas may correlate with the increased hormone dependency of their cells. The report of Vollenhoven et al. [40] demonstrated no difference in expression of the receptors between healthy women and patients treated with GnRH analogues. Also the studies on premenopausal women by Jakimiuk et al. [41] demonstrated similar expression of *α* and *β* receptors in myomas and in normal myometrium, with expression of receptor *α* persisting at a higher level than expression of receptor *β*. The results are consistent with our observations.

Even if some of the studies failed to differentiate between receptors *α* and *β*, it seems justified to conclude that such extensive differences in results are caused by individual, hormonal, and dynamic nature of the menstrual cycle and the individual character of disturbances in secretion of sex hormones during the perimenopausal period. The obtained results, manifesting a similar level in practically all groups comprised by the study, may suggest certain receptor stability in benign tumours. Slight differences in expression, observed in our study, may reflect heterogeneity of the tumours.

The receptor evaluated last involved the progesterone receptor. In our studies the receptor manifested certain specific distribution of expression in two age groups of patients: in small myomas expression of progesterone receptor was significantly higher than in the control groups, while in large myomas it was significantly lower than in the control groups and groups with small myomas.

Even if involvement of progesterone and its receptor in etiopathogenesis of myomas remains unquestionable [42, 43], the range of their involvement has not yet been fully recognized. Ishikawa et al. [44] observed evident myoma growth upon treatment in common with estradiol and progesterone. Upon administration of estradiol only the volume of the tumour did not increase and the authors concluded that estradiol induced expression of progesterone receptors in myomas, promoting in this way the pathological effect of progesterone. A similar mechanism was observed by Yamada et al. [45] in myoma cell cultures, where progesterone, alone or together with estradiol, induced the significant decrease in concentration of insulin-like growth factor-1 and the corresponding mRNA. On the other hand, no such changes could be induced with estradiol alone. Another problem is posed by the contradictory results obtained by certain authors, since, in studies using mifepristone and modulators of progesterone receptor, also the decreased amount of IGF-1 and of other growth factors were noted [45–47]. It seems that the results reflect heterogeneity of tumours or instability of receptors in the cell culture, the receptors which undergo decomposition within the first few days. This indicates that results of the studies should be cautiously interpreted.

Assuming that progesterone stimulates the growth of uterine myomas, it may be expected that it does not drastically affect the intensity of muscle cell proliferation. Also Zasławski et al. [48] detected no myometrial monthly cycle-dependent changes in expression of progesterone receptor, in contrast to some other available investigations. As mentioned earlier, proliferation of extracellular matrix participates in the growth of myomas [49]. An indirect evidence of the relationship between proliferation of extracellular matrix and progesterone level includes the therapeutic effects obtained upon the use of selective progesterone receptors [50, 51], inducing decrease in volume of the tumours.

It may be assumed that distribution of the receptor expression documented by the obtained results depends upon tumour heterogeneity and metabolic alterations taking place in the tumour along with its growth. At a certain stage of the tumour growth, such changes may decrease its sensitivity to progesterone.

Analysis of the obtained results, in view of the existing local feedback within the uterine myomas, should take into account the relationships linking GnRH-FSH-estrogens and GnRH-LH-progesterone. In the first relationship, any linkage between expressions of individual receptors seems to be impossible. Only in perimenopausal women the level of ER-*α* receptors was significantly higher with an almost undetectable expression of FSH receptor in the group. However, such low expression of FSH receptor reflected high plasma concentration of free FSH, noted in the early stage of the menopausal period and not the negative feedback reaction. The reports available [52] stress the importance of high expression of aromatase in the uterine myoma cells, accompanied by residual expression in normal tissues of the myometrium.

A similar system of statistically significant results was shaped within the relationship between GnRH-LH-progesterone level in groups of women with small myomas, independently of their age. Higher expression of LH receptor was accompanied by higher expression of progesterone receptor. In reproductive women, expression of GnRH receptor was additionally significantly elevated, while in perimenopausal women it was only slightly lower than in the control group. The above case may suggest a synergistically coupled action of progesterone receptor and LH receptor. In groups of patients with large myomas the obtained results were sufficiently inhomogeneous to allow for no cause-effect relationships. Despite continuously increasing knowledge on etiopathogenesis of myoma, it is still insufficient and further studies are indispensable to establish a platform for development of new approach to the treatment or modification of the already existing ones.

## 5. Conclusions

In the myometrium the level of GnRH and LH receptors increases with age, whereas the level of progesteron and both estrogen receptors decreases. In the uterine leiomyoma of reproductive women expression of GnRH, FSH, and LH receptors is higher than in the myometrium, regardless of myoma size. In uterine leiomyoma of perimenopausal women, expression of LH and estrogen *α* receptors is higher than in the myometrium, while expression of GnRH receptor is lower. FSH receptor expression was not observed. Progesteron receptor is highly expressed only in small uterine leiomyomas, whereas in large leiomyomas expression drops below the levels observed in the control group. Estrogen receptor *β* expression is independent of woman's age and size of the uterine leiomyoma.

## Figures and Tables

**Table 1:** Quantitative evaluation of receptors expression by immunohistochemical staining in myometrium and myomas of reproductive and perimenopausal age women.

Receptor	Reproductive age			Perimenopausal age		
	Control group	Small myomas	Large myomas	Control group	Small myomas	Large myomas
GnRH	98,7 ± 5,5	148,6 ± 8,9^b^	155,9 ± 10,8^b^	156,0 ± 9,0^a^	139,9 ± 9,6	116,6 ± 9,3^d,e,g^
FSH-R	61,7 ± 7,9	134,4 ± 9,1^b^	105,3 ± 7,9^b,c^	71,4 ± 6,7	—	—
LH-R	79,3 ± 5,9	184,5 ± 9,8^b^	162,3 ± 11,1^b,c^	109,8 ± 7,0^a^	142,9 ± 12,0^d,f^	144,8 ± 10,9^d^
ER-*α*	159,1 ± 12,4	160,7 ± 10,2	162,3 ± 10,8	98,7 ± 10,6^a^	142,7 ± 12,2^d^	140,6 ± 10,3^d,g^
ER-*β*	144,3 ± 10,1	150,2 ± 9,7	149,7 ± 9,5	111,7 ± 7,7^a^	116,4 ± 10,2^f^	122,2 ± 9,2^g^
PR	141,8 ± 8,7	182,4 ± 13,1^b^	102,5 ± 8,5^b,c^	91,9 ± 7,5^a^	145,9 ± 10,1^d,f^	63,5 ± 6,9^d,e,g^

The staining intensity was measured as described in methods. Data show an average optical density ± SD (see Section 2). Statistical significance was defined as a *P* < 0.05.

^a^Control groups; ^b^control group and any type of myoma for women in reproductive age; ^c^small and large myomas in reproductive age women; ^d^control group and any type of myoma for women in perimenopausal age; ^e^small and large myomas in perimenopausal age women; ^f^small myomas in both age groups; ^g^large myomas in both age groups.
